# “It’s way more than just writing a prescription”: A qualitative study of preferences for integrated versus non-integrated treatment models among individuals with opioid use disorder

**DOI:** 10.1186/s13722-021-00213-1

**Published:** 2021-01-27

**Authors:** Elizabeth C. Saunders, Sarah K. Moore, Olivia Walsh, Stephen A. Metcalf, Alan J. Budney, Patricia Cavazos-Rehg, Emily Scherer, Lisa A. Marsch

**Affiliations:** 1 Center for Technology and Behavioral Health, Geisel School of Medicine At Dartmouth College, 46 Centerra Parkway, Suite 301, Lebanon, NH 03766 USA; 2 Department of Psychiatry, Washington University School of Medicine, St. Louis, MO USA

**Keywords:** Patient preference, Opioid use disorder, Treatment model, Integrated treatment

## Abstract

**Background:**

Increasingly, treatment for opioid use disorder (OUD) is offered in integrated treatment models addressing both substance use and other health conditions within the same system. This often includes offering medications for OUD in general medical settings. It remains uncertain whether integrated OUD treatment models are preferred to non-integrated models, where treatment is provided within a distinct treatment system. This study aimed to explore preferences for integrated versus non-integrated treatment models among people with OUD and examine what factors may influence preferences.

**Methods:**

This qualitative study recruited participants (n = 40) through Craigslist advertisements and flyers posted in treatment programs across the United States. Participants were 18 years of age or older and scored a two or higher on the heroin or opioid pain reliever sections of the Tobacco, Alcohol, Prescription Medications, and Other Substances (TAPS) Tool. Each participant completed a demographic survey and a telephone interview. The interviews were coded and content analyzed.

**Results:**

While some participants preferred receiving OUD treatment from an integrated model in a general medical setting, the majority preferred non-integrated models. Some participants preferred integrated models in theory but expressed concerns about stigma and a lack of psychosocial services. Tradeoffs between integrated and non-integrated models were centered around patient values (desire for anonymity and personalization, fear of consequences), the characteristics of the provider and setting (convenience, perceived treatment effectiveness, access to services), and the patient-provider relationship (disclosure, trust, comfort, stigma).

**Conclusions:**

Among this sample of primarily White adults, preferences for non-integrated versus integrated OUD treatment were mixed. Perceived benefits of integrated models included convenience, potential for treatment personalization, and opportunity to extend established relationships with medical providers. Recommendations to make integrated treatment more patient-centered include facilitating access to psychosocial services, educating patients on privacy, individualizing treatment, and prioritizing the patient-provider relationship. This sample included very few minorities and thus findings may not be fully generalizable to the larger population of persons with OUD. Nonetheless, results suggest a need for expansion of both OUD treatment in specialty and general medical settings to ensure access to preferred treatment for all.

## Background

### Trends in opioid use disorder (OUD) treatment

With rising rates of opioid overdose deaths in the United States (US; [1]), more Americans are seeking treatment for opioid use disorder (OUD; [2, 3]). Despite an increase in admissions for OUD treatment in the US [3], there has been little expansion of specialty substance use treatment [4, 5]. Consequently, less than ten percent of American adults with an OUD received past-year treatment within a specialty substance use treatment program [6]. Though medications for opioid use disorder (MOUD), including methadone, buprenorphine, and naltrexone, are widely recognized as effective treatments for OUD [7–9], few patients receive these [10]. In 2017, only 38% of substance use treatment facilities nationwide offered MOUD [11]. As a result, more individuals are seeking treatment for substance use in non-specialty settings, including general medical settings [12, 13]. Providing treatment in non-specialty settings is critical to increase access to effective treatment, like MOUD, and reflects an important shift in the models of care for OUD and other substance use disorders in the US.

## Integrated versus non-integrated treatment models

Historically, care for behavioral and physical health conditions was offered in separate treatment systems [14–17]. However, recent legislation supported the expansion and integration of OUD and behavioral health treatment into general medical settings [14, 18–21]. Integrated treatment models systematically address both behavioral and physical health conditions within the same treatment system or program [22]. A range of integrated models for treating OUD have been implemented, often co-locating OUD treatment into primary care settings [23–26]. These models vary based on their level of primary care and behavioral health collaboration and the services offered [23, 25, 27, 28]. While some integrated programs offer MOUD only and refer patients for additional psychosocial services, others also provide psychosocial treatment services [24, 26, 29]. Evidence suggests that integrated treatment models within primary care are cost-effective, reduce treatment attrition, and may improve both health-related quality of life and satisfaction with treatment for patients with OUD [25, 28, 30–33]. Other settings, including pharmacies [34, 35], obstetrics and gynecology practices [36–38], and emergency departments [39] have also started to integrate MOUD and other OUD treatments.

## Patient preference for OUD treatment models

Although emerging evidence suggests that integrated treatment models are effective and associated with higher retention rates than non-integrated models of OUD treatment [28, 40], less is known about whether patients prefer integrated or non-integrated models. According to the Bastemeijer et al. taxonomy of patient values, patient preference is influenced by three primary considerations: 1) the patient’s values, including their desire for uniqueness and autonomy; 2) the characteristics of the provider valued by the patient, including their professionalism, responsiveness, and compassion; and 3) the patient-provider relationship, including their partnership and empowerment [41]. Consideration of patient preference is critical in the context of treating conditions that may necessitate long-term treatment, like OUD [42–44].

Few studies have examined patient preference for substance use disorder (SUD) treatment models [25]. A sample of patients at five clinics offering integrated primary care and behavioral healthcare indicated a moderate preference for integrated care on one survey item [45]. In another survey study, individuals on a web-based research panel were presented with vignettes describing either integrated or non-integrated treatment for behavioral health conditions. Among a subgroup of participants who screened positive for a SUD, 25% were willing to enter treatment when presented with the vignette describing usual, non-integrated care, while 37% were willing to enter integrated treatment [46]. These findings suggested substantial individual differences in preferences for treatment model that should be explored.

Several studies have assessed patient preference for integrated substance use and human immunodeficiency virus (HIV) treatment [47–50]. Overall, surveyed individuals generally expressed positive views regarding integrated versus non-integrated HIV and substance use treatment [47–50]. Patients appreciated the convenience of having a single team of providers [47, 48, 50] and felt their integrated care was comprehensive and highly personalized [48, 50]. Despite this, some patients preferred receiving substance use treatment from providers in specialty clinics not associated with their HIV care [48, 50] because of concerns that HIV providers may lack training and knowledge about treating substance use [48] and because of the additional structure offered in specialty substance use treatment programs [48, 50].

Few studies have assessed preferences for integrated OUD treatment located in settings other than HIV clinics, such as primary care, emergency departments, or pharmacies. In a cross-sectional study of people who used opioids at a syringe services program, 62% (n = 49) were willing to initiate buprenorphine treatment in a specialty substance use treatment program while 59% (n = 47) were willing to initiate in a primary care clinic [51]. Two qualitative studies found that patients receiving buprenorphine in primary care settings generally reported being satisfied with their care [52, 53]. These patients appreciated the convenience of being treated for both physical health and substance use concerns in one setting and felt that the primary care providers and staff were courteous and respectful [52]. While these studies examined satisfaction and willingness to initiate care among patients with OUD, it remains uncertain whether people with OUD prefer integrated versus non-integrated treatment models and what factors may influence preferences and perceptions for these treatment models.

## Aims and objectives

The goals of the present study were to examine preferences for integrated versus non-integrated treatment models among adults with OUD and to explore the factors influencing these preferences. Qualitative interviews were conducted to provide an in-depth understanding of factors shaping preferences for treatment models, as well as participant preference for different MOUD formulations [54]. This article focuses on the results from the analysis exploring preferences for OUD treatment models, which the authors planned to use to refine a survey instrument to examine preferences for OUD treatment in a subsequent study.

## Methods

### Design

Qualitative interviews were conducted by telephone with adults reporting non-medical use of opioid pain relievers, heroin, and/or fentanyl. The semi-structured interview guide (Additional file 1) was developed with input from experts in qualitative methodology and OUD treatment, after completing a literature review of studies examining OUD treatment preferences. This interview guide was not pilot tested but was iteratively revised after conducting the first two interviews based on participants’ understanding of the prompts. Interviews focused on participants’ opioid use, experiences with treatment for OUD, and preferences for MOUD formulation and integrated treatment models. Though findings were organized by the domains from the Bastemeijer et al. taxonomy of patient values [41], participants were first asked more open-ended and exploratory questions about their thoughts and preferences surrounding treatment models. If participants were not forthcoming with their thoughts, the interviewer followed up with questions regarding the participants’ prior experiences with general medical providers and level of comfort discussing substance use with general medical providers. Each participant also completed a brief survey created for this study, which collected data on participant demographics, opioid use, and history of receiving OUD treatment. No repeat interviews were conducted. The Dartmouth College Committee for the Protection of Human Subjects approved all study materials and methods.

## Participants and setting

Forty participants completed interviews in February and March of 2018. Thirty were recruited through Craiglist, and ten were recruited through flyers posted in substance use treatment programs. Individuals under 18 years of age were excluded. Other inclusion criteria included English-language proficiency, current US residence, and a score of two or higher on the heroin and/or opioid pain reliever sections of the Tobacco, Alcohol, Prescription Medications, and Other Substances (TAPS) Tool [55, 56], suggesting a past-year OUD diagnosis. Once determined eligible, no participants refused to participate or dropped out.

Participants were purposively recruited to ensure diversity in geographic location and MOUD experience. Because MOUD experience may influence OUD treatment model preference, the researchers purposively recruited individuals with and without MOUD experience through advertisements posted in the “Help Wanted-Gig” section of Craigslist, an online classifieds website. These advertisements were posted in nine regions of the US, including urban and rural regions in the Northeast, Southeast, Midwest, Northwest, and Southwest known to have high rates of opioid overdose [57]. Flyers were also posted in six specialty substance use treatment programs affiliated with the National Drug Abuse Treatment Clinical Trials Network in the states of New York, Texas, Washington, Oregon, and Ohio to recruit individuals receiving MOUD treatment. These programs were all specialty substance use treatment programs. The flyers and advertisements were posted in English. We planned to recruit up to 40 participants and continue interviews until thematic saturation was reached with the added proviso that saturation was not understood as an event but an incremental assessment of the substantiation of themes [58].

## Procedures

Participants contacted the research team by phone or email to schedule an interview. All interviews were conducted by the first author (ECS), a female health policy and clinical practice doctoral candidate. This interviewer had received doctoral-level training on qualitative interviewing and also had experience conducting interviews about opioid and other substance use in several previous studies [59–61]. The interviewer had no relationships with any participants prior to their involvement in the study. When participants were introduced to the study, the researcher introduced herself as part of a research team studying treatment for OUD. She explained this study was part of her doctoral research and informed participants that the primary purpose was to learn about participants’ experiences and preferences for different treatment for OUD. The interviewer explained that there were no right answers to any of the study questions and told participants that the research team hoped to learn from their experiences and opinions.

After describing the study, the interviewer read aloud the study information sheet and answered questions about the study. Once participants provided oral consent, they were screened to determine eligibility. Because the study recruited individuals from across the US, all interviews were conducted by telephone. Usually participants were alone while completing the interviews, though in several interviews the interviewer became aware of the presence of other household members. Participants completed the brief demographic survey, followed by the semi-structured interview. To facilitate the interview, participants who were willing to share their email address were sent a Treatment Model Comparison Chart (Additional file 2) created for the study, which described integrated versus non-integrated treatment models. If participants did not wish to share an email address, the interviewer read aloud the information contained in this chart.

Interviews were audio recorded. Recordings were uploaded to a secure electronic folder hosted by Dartmouth College and were only identified with the study identification number. The interviewer also wrote brief field notes on a spreadsheet after each interview to facilitate recall of contextual variables during analysis and interpretation. Though phone numbers and a pseudonym were also collected for each participant to facilitate scheduling, these were deleted once all interviews were complete. As compensation for their time, participants were sent a $40 gift card by email, postal mail, or text message.

## Analysis

All interviews were transcribed verbatim. Half of the interviews were transcribed by a third-party transcription service, and the other half were transcribed by members of the research team using Express Scribe transcription software [62]. Each transcript was reviewed by at least one member of the research team. Transcriptions were not returned to participants for comment or corrections. After transcription, any potentially identifying information, such as person or street names, was removed from every transcript. Transcripts were then analyzed using a directed content analytic approach [63–65]. A preliminary code list was developed deductively based on the topics and domains covered in the study interview guide. Initial coding categories were determined based on the interview guide, which was created using the existing literature about patient preference for OUD treatment. Once the preliminary code list was developed by one analyst (ECS), transcripts were uploaded to Atlas.ti [66] for analysis. First-cycle coding was completed by two analysts (ECS, SKM), who initially coded two transcripts collaboratively. After reviewing each transcript, all text was coded using the predetermined codes. Text that did not fit into the pre-determined codelist was marked. The analysts discussed these marked sections and emerging coding categories weekly. New codes were then inductively generated, and the code list was revised. The analysts then collectively analyzed two additional transcripts, until reaching consensus that no new categories were conceptualized. At this point, the analysts independently coded the remaining 36 transcripts, meeting weekly to discuss coding and resolving through discussion.

The subtheme analysis was conducted by four analysts (ECS, SKM, OW, SAM). Coded text segments were exported to Microsoft Word. For every code, two analysts reviewed the coded passages line by line. Text segments were grouped based on emerging themes and subthemes. The analysis team met regularly to discuss and compare findings. Text segments stating preference for integrated versus non-integrated treatment were counted quantitatively by two analysts working independently who met and compared findings weekly (ECS, OW). Throughout the analysis, discrepancies were resolved through discussion. The Bastemeijer et al. taxonomy [41] was used as a heuristic device to conceptualize the findings from this analysis by examining advantages and disadvantages of integrated versus non-integrated treatment models. This taxonomy includes three primary considerations that influence patient preference: patient values, characteristics of the provider, and the patient-provider relationship [41]. In addition to provider characteristics, characteristics of the treatment setting emerged as an additional important theme for participants. The Bastemeijer et al. taxonomy was thus modified to include this theme after completion of the subtheme analysis. Participants did not provide feedback on the findings.

## Results

### Participant characteristics

Participants’ average age was 36.5 (standard deviation [SD] = 10.9) years. Forty percent of participants (n = 16) were female, while 60.0% (n = 24) were male. The majority of participants were White (n = 36; 90.0%), with only four Black or African American participants (n = 4; 10.0%). Eighty percent of participants were not Hispanic or Latino (n = 32). Based on the National Center for Health Statistics Classification [67], 18 participants (45.0%) resided in large metropolitan regions, 13 (32.5%) in medium metropolitan regions, and 8 (20.0%) in micropolitan or non-core regions of the US. Participants’ average TAPS total score was 4.8 (SD = 1.6) points (range: 2–6 points). While all participants reported use of non-prescribed opioid pain relievers, 27 (67.5%) also reported use of heroin and/or non-prescript8ion fentanyl. The majority of participants (n = 36; 90.0%) had received some form of treatment for their OUD during their lifetime, and over half had been prescribed MOUD (n = 24; 60.0%). Among the 36 participants who had received treatment, ten (27.8%) reported receiving treatment from an integrated treatment program. Of the ten participants currently prescribed MOUD, two participants were currently receiving MOUD from an integrated treatment program. Additional details about participants’ demographic and opioid use characteristics are available in Table 1.

**Table 1:** Participant demographics, opioid use characteristics, and treatment experiences

	Participants (n = 40)
*Demographic characteristics*	
Age m (sd)	36.5 (10.9) years
Gender n(%)	
Male	24 (60.0%)
Female	16 (40.0%)
Non-binary	0 (0.0%)
Race n(%)	
White	36 (90.0%)
African American/Black	4 (10.0%)
Ethnicity n(%)	
Hispanic or Latino	8 (20.0%)
Not Hispanic or Latino	32 (80.0%)
Regions of residence n(%)	
Midwest	5 (12.5%)
Northeast	10 (25.0%)
Northwest	5 (12.5%)
Southeast	11 (27.5%)
Southwest	9 (22.5%)
Highest level of education n(%)	
Less than high school	4 (10.0%)
High school degree/GED	12 (30.0%)
Some college	11 (27.5%)
Associate’s degree	4 (10.0%)
Bachelor’s degree	7 (17.5%)
Master’s degree	1 (2.5%)
Trade school	1 (2.5%)
*Opioid use and treatment characteristics*	
Recency of opioid use n(%)	
Past week	10 (25.0%)
Past month	6 (15.0%)
Past 6 months	9 (22.5%)
More than 6 months	15 (37.5%)
Treatment experiences n(%)	
Current/past OUD treatment experience	36 (90.0%)
Current/past MOUD prescription	24 (60.0%)

## Preferences for integrated versus non-integrated treatment models

The qualitative section of the interviews was 45 to 60 min in length. When asked about their preference for receiving integrated versus non-integrated care, nine participants preferred integrated models, sixteen preferred non-integrated models, and six were more uncertain. The six uncertain participants preferred the idea of integrating OUD and general medical care in theory but had concerns that the reality of integrated care may be stigmatizing or not provide necessary support or services. One participant explained, “I think [integrated care models] would save people who are suffering with an addiction a lot of stress, if there wasn’t such a stigma, if your primary care doctor didn’t look at you like a scumbag for having a substance abuse issue” (Identification Number: 101, Gender: Male, Age: 31 years). These tradeoffs were reflected in participants’ discussion of perceived advantages and disadvantages of integrated treatment models (Table 2).

**Table 2:** Perceived advantages and disadvantages of integrated treatment models: Emergent themes and representative quotes organized using the Bastemeijer et al. (2017) taxonomy

	Factors influencing preferences for:	
Theme	Integrated models	Non-integrated models
*Patient values*		
Privacy and confidentiality	“When you go to a treatment program and stuff, it’s public. They’re not supposed to say anything, but it kinda becomes a public record. I mean, word gets out quick.” -140, Male, 40 years	“I would like to go to a private facility that nobody knew about… and they don’t share information with anybody else. I just want to focus on getting better, as opposed to what else is going on with this information.”-135, Male, 30 years
Fear of consequences	–	“My struggle with addiction isn’t something that I readily share with [general medical providers]. One, because I want the doctor to not ever prescribe me pills again. And two, especially now that our entire healthcare system has moved to electronics, I don’t really have any state privacy.” -138, Male, 33 years
Individualization	“The cool part about doing everything with him [general medical provider] would be that it would be really personal.” –105, Female, 31 years	–
*Characteristics of setting and provide*r		
Convenience Ease of access	“I mean, he [primary care doctor] is right in the same town that I live in, and it just would have been convenient.” –116, Female, 29 years	–
Speed of access	“I wouldn’t have to wait to get approval to get seen.” –105, Female, 31 years	“I think you’d have to make a doctor’s appointment, and sometimes you have to wait a long time before you can see a doctor. I think that would be a con [of integrated models] because I know when I go to see my doctor, I have to wait a frickin’ month before I can see him.” –107, Male, 52 years
Cost	“I think that [integrated treatment] would help a lot of people. It would probably help a lot of people because it costs moey to get clean like that [non-integrated treatment models]… Like, going to the Suboxone doctor’s like hundreds and hundreds of dollars a month.” –113, Female, 38 years	–
Effectiveness at treating OUD	–	“I don’t know if I would necessarily talk to my primary care doctor [about my OUD] because I don’t think it would be beneficial at all.” –104, Female, 25 years
Provider training in treating substance use	–	“To get the license to prescribe [buprenorphine], apparently, it’s a very, very short course. There’s a lot to addiction. I mean, it’s a very complex thing. And it’s just not a one plus one is two… I don’t think they [general medical providers] have the amount of education for it.” –112, Male, 34 years
Access to services		
Counseling	–	“A [general medical provider] can probably do the medical part, but I still think a person still needs that treatment.” -131, Female, 36 years
Peer support	“I’d rather do [integrated treatment]. If you’re an addict and you’re trying to stay clean, but you have to go somewhere every day where there’s a thousand other addicts there, that’s not good for people.” –115, Female, 35 years	“I would probably go to a treatment program, and maybe if I had some other people to talk to and I could talk to people about what I’m going through, I wouldn’t feel as bad because I would hear their stories and stuff.” –120, Female, 25 years
Structure and support	–	“The negative part about going to [a general medical provider] would be a lack of knowledge or tools to help me, and not having other people there who can support me. I would fully rely on him for treatment.” –105, Female, 31 years
Patient-provider relationship		
Pre-existing relationship	“When it comes to life or death, wouldn’t you want somebody, like your doctor, that you’ve met over and over again? That you felt a connection with?” –119, Male, 42 years	“I went to [a general medical provider], and he was asking me some questions about do I use. And I really was like, ‘I don’t want to tell this man that!’ ‘Cause people look at you differently when they have to take care of you then. It seems like he did after I told him.” –-131, Female, 36 years
Past experiences of disclosure	“He [general medical provider] knows all about my history, and that’s something that was really important to me in a doctor, to find somebody who I could be honest with, because you know, what good is it if you can’t be.” –116, Female, 29 years	“She treated me differently once she found out I was an addict. I felt like everything shifted.” –115, Female, 35 years
Trust	“They’re [general medical providers] trusted, and I already know who they are. It’s a good way, going with whatever the doctor that you have.” –-132, Male, 28 years	“I don’t even trust [general medical providers] anymore. Like after everything I’ve been through, I don’t even trust doctors. I feel like it’s all a scam. I feel like they’re just out to make money… That’s it. They don’t really want to help you.” -113, Female, 38 years
Comfort discussing substance use	“I feel comfortable talking with him [general medical provider] about everything. And the people at the clinic.” -110, Female, 32 years	“If I was put in a situation where I would have to go talk to my doctor about my drug usage, I definitely would not feel comfortable with that.” –127, Male, 33 years
Compassion	“They [general medical providers] just make you feel like you’re a person and not just like a frickin’ junk box.” –110, Female, 32 years	“Most people in the medical field, a lot of them don’t show that caring… It’s just a cattle call.” –102, Male, 57 years

### Perceived advantages and disadvantages of integrated treatment models: Patient values

#### Patient values: Advantages of integrated treatment models

The opportunity for treatment personalization was valued as an advantage of integrated treatment. Some participants described encountering a “one-size-fits-all” (122, Male, 37 years) approach to treating OUD at specialty substance use treatment programs.

“You go into a place at the rehab, or you go to a place that deals with substance abuse… Like the second you walk in, they automatically label you as every other addict in the world… They don’t individualize [treatment]. Especially, like with group counseling. They’re just like, ‘OK, there’s 20 people in this room. So you all should deal with it like this.’ That’s not how we work. It doesn’t work for everyone” (104, Female, 25 years).

Several participants believed that integrated models of care may have less rigid treatment plans and valued the opportunity to individualize treatment based on their own desires and needs. A participant described her experience in an integrated treatment setting, explaining, “[My primary care provider] was willing to work with me on a more individual approach… She allowed me, when I was in outpatient, she was like, ‘So instead of these four AA meetings that you have to sign for, you can go to two, and then there’s also this SMART Recovery’” (139, Female, 24 years).

#### Patient values: disadvantages of integrated treatment models

A desire for anonymity was expressed by participants and was generally viewed as a disadvantage of integrated treatment for OUD. Participants often felt that receiving treatment integrated into general medical settings was less private and confidential than non-integrated treatment. For some, these concerns centered on the number of providers encountered in general medical settings. “Anonymity is a big thing… When you go see a doctor, a nurse goes through everything first, before the doctor comes in. So now you got the nurse knowing what you are in here for. When you’re a drug addict or an alcoholic, it’s kind of a secret. I don’t think too many people would go for [integrated treatment]” (107, Male, 52 years). These concerns appeared heightened by dual relationships with general medical providers for a few rural patients. “I live in such a small town. Everybody knows everybody’s business. Let me just put it this way: One of the medical attendants is actually my neighbor. So, I know she knows everything that’s within my chart” (135, Male, 30 years). Participants also had concerns that their medical records were accessible by too many individuals in general medical settings. “Now that our entire healthcare system has moved to electronics, I don’t really have any state privacy. So honestly in the past, I’ve never felt comfortable sharing these things with a primary care doctor” (138, Male, 33 years). Though participants generally expressed awareness that treatment received at specialty substance use treatment programs was protected by federal regulations, there was uncertainty regarding the rules for protecting OUD treatment information within general medical settings. “As far as therapists go, they have the whole confidentiality oath that they have to go ahead and respect. And I don’t know if doctors are the same. I mean, I know they have the Hippocratic Oath, but that’s another thing” (127, Male, 33 years). Only one participant thought integrated models may be more private than non-integrated models, explaining that “when you go to a [substance use] treatment program, it’s public. They’re not supposed to say anything, but it kinda becomes a public record. I mean, word gets out quick” (140, Male, 40 years).

Participants also worried about the personal consequences of disclosing their OUD in general medical settings, specifically expressing concerns about being denied medications (e.g., “they’ll push that red button on the computer, and you’ll never get an opiate for the rest of your life” [119, Male, 42 years]), losing insurance coverage (e.g., “a lot of insurance companies might use that information adversely to me” [135, Male, 30 years]), or being reported to the legal system (e.g., “that’s why a lot of people don’t ask for help, because of the legal issues” [106, Female, 29 years]). One participant summarized these concerns, explaining “[Integrated models] would scare people off, because [people] don’t want to go ahead and get their medical plan messed up or they probably don’t want to be reported… I wouldn’t want to be in talking to any authority that’s going to be able to affect my life by being honest” (127, Male, 33 years). In multiple cases, participants described experiences in which their medical treatment was altered after disclosure of non-prescribed opioid use, including treatment for migraine, mental health conditions, and chronic pain.

“A specialty setting is probably better, I would think… I went in to [a general medical setting] to get headache medicine refilled… And they treated me like I was a criminal when I went in there. They asked me to do a drug test, which I wasn’t opposed to, I didn’t mind, but she also asked me if I abuse my medicine, which it can’t be abused… I never got my headache medicine that she still never called in. Had to fight with them for a week, and then I never went back” (114, Female, 27 years).

### Perceived advantages and disadvantages of integrated treatment models: characteristics of the provider and setting

#### Characteristics of the provider and setting: advantages of integrated treatment models

Unanimously, participants thought that integrated care models were “a lot more convenient” (134, Female, 53 years) than non-integrated models, as they were often geographically closer, faster to access, and less expensive than specialty substance use treatment programs. Some participants appreciated being able to receive treatment for their substance use in the same setting as they received other health care. Participants described encountering lengthy waiting lists and delays in receiving insurance approval prior to engaging in OUD treatment in non-integrated settings. Participants, therefore, felt that receiving OUD treatment in a general medical setting would expedite this process. “[Integrated treatment] is good so that you have access, but you don’t have to wait for help” (106, Female, 29 years). Similarly, several participants thought integrated models might reduce the costs of treatment for patients. “The primary care doctor would have been such a great thing to do instead of having to go to a specialist that charges more money” (113, Female, 38 years). No participants perceived non-integrated models to be more convenient, cheaper, or easier to access.

#### Characteristics of the provider and setting: Disadvantages of integrated treatment models

Though convenience was a distinct advantage for integrated models, participants reported significant doubts regarding the effectiveness of integrated models on treating OUD. More than half of participants expressed uncertainties about general medical providers’ abilities to treat opioid use successfully. “I guess if I had to choose… I think I would still want to go to a [non-integrated model] to get help because I feel like it’s probably the most effective way to get treatment because they have these programs that are specifically designed for you and your addiction” (105, Female, 31 years).

A quarter of participants thought that general medical providers are not qualified to treat OUD because “they’re not educated on it” (115, Female, 35 years). Participants believed most general medical providers had little training on substance use or OUD. “I’ve heard that most doctors don’t have much knowledge with addiction. So, I don’t feel like they should be prescribing [MOUD] unless they’ve gone to school for quite a while, and they have a lot of knowledge about how to go about treating addiction. It’s way more than just writing a prescription” (137, Female, 45 years). Several participants felt that asking a general medical provider to treat OUD, rather than a psychiatrist, psychologist, or counselor, was “asking to fail” (111, Male, 30 years) and thought general medical providers “don’t have any business prescribing [MOUD] because that’s not their specialty” (109, Female, 32 years). Many participants therefore expressed a desire to receive treatment from a provider who specialized in treating substance use and had more in-depth training and knowledge about OUD.

“They [a primary care physician] deal with such a wide variety of illnesses that, I don't want to say that they couldn't handle it, but what I have is severe. So that's why I prefer a [specialist]. But, I mean that's what primary care was, they were Google before Google” (111, Male, 30 years).

A lack of time in general medical settings was identified as potentially reducing the effectiveness of integrated treatment models. “My primary care doctor has no time to do the Suboxone. It’s not fair to him” (124, Male, 65 years).

Others doubted that the context of general medical settings would be conducive to providing effective treatment, perceiving these settings to lack structure and support. Though participants desired the opportunity to individualize their treatment, they emphasized the importance of easy access to psychosocial services, if desired. Concerns, therefore, that integrated models may fall short by not facilitating access to counseling or peer support were common.

“I personally haven’t heard of [general medical providers] prescribing Suboxone in relation with groups or continuing care at all. The ones that you go to, they kind of put that on you, but if there was a silver bullet to fix all this shit, it’s not Suboxone by itself provided by a prescriber. That’s wonderful that they can do that, but there needs to be other support. If they’re not providing it, they should facilitate it being available, if nothing else” (136, Male, 34 years).

While many participants desired peer support, one participant thought that reduced contact with other people with OUD was an advantage because of the decreased risk of interaction with people who were actively using.

### Perceived advantages and disadvantages of integrated treatment models: Patient-provider relationship

#### Patient-provider relationship: Advantages of integrated treatment models

Participants who viewed having a pre-existing relationship with a general medical provider as an advantage described having strong, positive relationships with general medical providers. This was a facilitator of preference for integrated treatment, as several of these participants wanted to expand this positive relationship to include treatment for their OUD. “It’s really hard to see a medical professional in your most addicted state, and they don’t know you at all. They don’t know your body, and they don’t have any basis of comparison for before you had this addiction. So yeah, it would have been great if my primary care was able to do it” (116, Female, 29 years). Many of these participants felt comfortable discussing their substance use with general medical providers. “I do feel comfortable talking to my [primary care provider]. Actually, she’s the best doctor I’ve ever had… The first thing she does when she walks in, is she shakes your hand. You don’t have a lot of doctors do that… I thought that was unusual and from then on, I’ve trusted her decision-making” (118, Male, 33 years).

#### Patient-provider relationship: Disadvantages of integrated treatment models

Conversely, other participants were not comfortable discussing their substance use with general medical providers and described negative relationships with providers. These participants thought that general medical doctors often did not approach substance use in a sensitive manner or expressed judgmental attitudes. “Any time that I’ve gone to the doctor or even gone to the ER or the hospital or anything like that, they’re literally like, ‘Do you have any existing medical conditions? Do you have diabetes? Oh, do you smoke crack on the side?’ Like no, that is not how we should approach that at all” (104, Female, 25 years). Though one participant viewed general medical providers as more compassionate than providers in specialty settings, other participants expressed frustration with a lack of compassion and connection. “We don’t treat human beings like human beings anymore. That’s what drug addicts need. They need people that care” (102, Male, 57 years). Providers working in specialty settings were perceived to be “just a lot nicer” (125, Male, 35 years) and have more knowledge and compassion when discussing substance use.

Past experiences of disclosure in general medical settings often impacted participants’ perceptions of receiving treatment in integrated models. Participants described experiencing stigma and judgement after confiding in general medical providers, explaining that these providers then treated them like a “scumbag” (101, Male, 31 years) or a “criminal” (114, Female, 27 years; 127, Male, 33 years) after learning of their opioid use. “My [primary care provider] treats me differently once she found out I was an addict. I feel like everything shifted” (115, Female, 35 years). Seven participants described specific situations in which general medical providers treated them differently after disclosure of substance use.

“One time I went into a pain management specialist, and I told him that I was having withdrawals. I was honest with him that I had been abusing opiates, and I requested Suboxone. And he was a prescriber of Suboxone… I had told him that I had been using oxycodone. Because I had been. And my supply had run out, and I couldn’t find it, and I did use heroin a few times, in that span, that run. My urine test came back positive for opiates and not for oxycodone, so because I was not truthful in his words, he just kind of told me to beat it, which I thought was just cruel” (101, Male, 31 years).

Fear of judgement, often stemming from these past experiences of disclosure, was a major concern surrounding integrated care models. “There’s such a taboo, especially around a lot of behaviors, whether they’re sexual or drug related. It is hard to find a [medical provider] that isn’t kind of judgmental about that sort of thing” (116, Female 29 years).

As a result of previous negative experiences and a belief that “doctors have a lot of accountability in this epidemic” (101, Male, 31 years), five participants lacked trust in general medical providers. “For many years, primary care doctors were prescribing a lot of opioids. So, I don’t know if there’s a lot of people that trust them. A lot of those doctors, they cashed in, and they’ve gone on. So, I don’t know” (108, Male, 36 years). These participants did not trust that general medical providers would sincerely look out for their wellbeing, expressing that “I feel like doctors are out to make money. That’s it. They don’t really want to help you” (113, Female, 38 years). Aside from concerns about trusting providers, participants doubted that general medical providers would trust a person with an OUD. “There’s no level of trust inherent in a person who is an addict. So, an addict going to a doctor and trying to plead their case for whatever. I just feel like it carries a lot less weight when you have a substance abuse issue” (101, Male, 31 years). As a response to this mutual mistrust, participants worried that their other medical concerns may be overshadowed by their substance use and ignored by their medical providers.

## Discussion

Across the US, treatment for OUD has been increasingly integrated into general medical settings to rapidly expand capacity in the wake of the opioid overdose epidemic [68]. In response to this epidemic and lack of capacity in specialty treatment settings [4, 5], an increasing number of individuals are accessing critical treatment for OUD in non-specialty settings, including general medical settings [12, 13]. These integrated programs are critical to reducing the rate of overdose deaths in the US by expanding access to life-saving MOUD, which is still underutilized in specialty settings [10, 11]. Emerging evidence also suggests integrated MOUD and primary care models can improve health-related quality of life and treatment retention for patients with OUD [25, 28, 30–33]. Despite the benefits of integrated treatment, little previous research has examined patient preferences for integrated versus non-integrated OUD treatment models. Results of this exploratory, qualitative study suggest that although some participants would theoretically prefer receiving OUD treatment integrated in general medical settings, others prefer non-integrated treatment models. Participants considered the advantages and disadvantages of integrated models in terms of their own values (desire for anonymity, fear of consequences, a desire for personalizing treatment), characteristics of the provider and setting (convenience, perceived effectiveness, and access to services), and their relationship with medical providers (navigating a pre-existing relationship, past experiences of disclosure, trust, comfort, stigma, and compassion). These values, opinions about the provider and setting, and patient-provider relationships were substantially influenced by participants’ prior experiences with OUD treatment and the general medical system.

## Patient values and OUD treatment model

Participants valued a sense of anonymity when receiving OUD treatment and expressed concerns about the privacy of care offered within integrated treatment models. These concerns were amplified by the ubiquitous use of electronic health records in general medical settings. Relatedly, participants dually considered the consequences of disclosing their OUD in terms of the impact on their treatment and on other facets of their life, if this information about their OUD were to be shared without their permission. Research suggesting that substance-related records are often accessible to providers within integrated treatment settings, despite the 42 CRF Part 2 regulations, gives credence to participants’ privacy concerns [69]. At a policy level, clarifying rules and regulations protecting substance use treatment information within integrated treatment models may be critical to engaging patients. Integrated treatment programs should also consider educating patients regarding how their OUD treatment information will be protected within the program’s health records and which staff will have access to this information. This may be particularly important in rural regions, where dual relationships between patients and providers are more common [70–72].

The potential to personalize treatment was considered an important advantage to integrated models of OUD treatment. Participants perceived that general medical settings may have less rigid OUD treatment pathways. Patients seeking SUD treatment have widely expressed a desire to be involved in planning their treatment [73–75]. According to a recent review, forty-six studies have explored individualized treatment approaches [76]. Individualized approaches typically involved 1) a needs assessment and treatment planning to match patients to treatments, 2) delivery of treatment according to patient preferences and needs, or 3) adapting treatment to patients’ unique assets and challenges. Integrated programs could consider adopting clinical approaches identified in this review that supported the development of individualized care, including encouraging patients to share preferences, establishing caring relationships with patients, and recommending a flexible continuum of care.

## Provider characterisics and setting

In contrast, several characteristics of general medical providers and practices were considered disadvantages to receiving care through an integrated treatment model. Though evidence demonstrates that MOUD with minimal counseling can be highly efficacious [77–79], participants still worried that many general medical settings would not offer psychosocial services or facilitate access successfully. The components of OUD treatment offered in general medical settings do vary widely across practices [24, 80]. Treatment guidelines recommend that OUD treatment integrated into primary care or general medical settings should include counseling and other psychosocial services in addition to MOUD [81, 82]. These recommendations suggest that integrated OUD treatment in general medical settings contain four treatment components: 1) MOUD; 2) psychosocial services; 3) integrated care for physical and psychiatric problems; and 4) education and outreach [24]. Despite these recommendations, many general medical practices struggle to provide these services due to a lack of time, staff, and financing [80]. Implementing specific integrated models, like the Collaborative Care or the Embedded Behavioral Specialists models that co-locate a care manager or behavioral health clinician onsite, could facilitate access to psychosocial services [83, 84]. In regions where psychosocial services may be less accessible, general medical settings providing OUD treatment should consider leveraging novel technologies to provide access to patients, such as telepsychiatry [85, 86] or mobile health (mhealth) applications [87–90].

Participants also expressed doubts about the ability of general medical providers to effectively treat their OUD, noting concerns about training. These concerns are often mirrored by general medical providers, who have widely reported feeling unprepared to treat substance use disorders [60, 91–95]. An opportunity to increase training and education exists, as general medical providers in one survey study strongly believed treatment for OUD could be effective and supported improving education and training [96]. General medical practices that plan to integrate treatment for OUD should provide training beyond the buprenorphine waiver training, including allocating time for providers to participate in training and mentoring activities, such as the Provider Clinical Support System for Medication-Assisted Treatment (PCSS-MAT; [97], learning collaboratives [98], or Project Echo [99, 100].

In opposition to the perceived disadvantages of integrated treatment, participants unanimously agreed that integrated treatment was more convenient and less costly than non-integrated treatment. In studies exploring preferences for integrated substance use and HIV care, patients similarly valued having both health conditions treated by a single set of providers in one setting [47, 48, 50]. Convenience and cost are well-established barriers to receiving specialty substance use treatment [6, 76, 101]. Some rural participants described lengthy commutes to obtain specialty substance use treatment, so the geographic proximity of general medical settings was an important advantage of integrated models. People with OUD residing in rural settings are less likely to obtain specialty substance use treatment [6], so integrated models may be critically important to providing access to care in these regions.

## Patient-provider relationship

The patient-provider relationship was another factor that strongly influenced preferences for integrated OUD treatment models. Depending on each participant’s individual experiences and values, participants viewed this relationship as either an advantage or disadvantage of receiving treatment integrated in general medical settings. For participants with strong existing connections with general medical providers, this established relationship was viewed as an advantage. Research suggests that people generally feel more comfortable disclosing substance use to those with whom they have a long and trusting relationship [102], so it is therefore not surprising that this relationship may impact participants’ preferences for addressing their opioid use with a general medical provider.

For other participants, previous experiences of stigma and judgement from general medical providers contributed to an absence of trust, feelings of shame, and discomfort discussing their opioid use in general medical settings. These experiences of stigma in medical settings are barriers to utilizating health care in general [103–105] and may likewise impact access to OUD treatment in integrated settings. For patients who do seek treatment despite past experiences of judgement or stigma, these experiences can make patients more vigilant and magnify any negative interactions with medical providers in the present [106]. The patient-provider relationship also has potential effects on participants’ views of provider competence and their concerns about privacy. Strong and trusting patient-provider relationships can reduce the impact of privacy concerns [107, 108], so the negative relationships described by some patients may intensify these privacy concerns. In addition, patients rate emphatic providers as more competent [109, 110]. Therefore, considering interventions and methods to improve the patient-provider relationship may support patient engagement in integrated treatment.

Aside from viewing providers who specialize in addiction medicine as more knowledgeable and effective at treating OUD, the majority of participants also thought specialists would be less judgmental and more understanding. This opinion echoes findings of a recent qualitative study [105] in which people who injected drugs thought addiction medicine specialists were more empathetic and helpful than general medical providers. A review of studies examining attitudes toward patients with OUD among health providers found that stigma and negative attitudes are commonly held in medical settings [111]. Improving the patient-provider relationship by mitigating stigma and judgement may be critical to engaging people with OUD in integrated models of treatment. Effective interventions to reduce stigma in medical settings include communicating positive stories about people with OUD and improving medical provider education about SUDs [112, 113]. The adoption of person-first language (e.g., person with an opioid use disorder) may also help reduce stigma in general medical settings [114–117].

## Limitations

This study recruited a relatively small, self-selected sample of people who were predominately White and not Hispanic or Latino. The lack of racial and ethnic diversity among participants is a major limitation of this study. Studies have clearly documented disparities in OUD treatment access among people of color in the US [118–120]. In addition, structural racism and experiences of racial discrimination in healthcare settings may impact individuals’ experiences with treatment [121–123] and thereby influence their preferences for OUD treatment. Moreover, recruitment materials and interviews were all in English, hence excluding individuals without English language proficiency, who may face additional barriers and have different experiences with treatment than individuals who speak English. To better understand preference for treatment models, future research must include more participants of color and participants without English language proficiency.

Using a TAPS cutoff of two or higher likely excluded some individuals with less severe opioid use. Participants may represent a more severe sample than the general population of individuals with OUD in the US. The attitudes and preferences of study participants could be systematically different from people who were not interested in participating, as participants may have had stronger opinions about this topic than those individuals who did not participate. Preferences and perceptions are also influenced by past experiences. While some participants had experiences receiving OUD treatment, others had never received treatment. This study was unable to systematically examine the impact of past treatment experiences. Additionally, among those who had received treatment, participants’ experiences with each treatment model may have varied substantially, which could also impact preference. More participants were currently receiving MOUD from non-integrated than integrated programs. It is possible that individuals currently receiving treatment in a non-integrated setting may prefer this setting, so future research should recruit from integrated programs in addition to non-integrated programs in order to include more participants currently receiving integrated treatment.”

Last, nine participants in this study were unable to articulate a preference for integrated versus non-integrated treatment models. While some of these participants may indeed not have a strong preference, a lack of understanding regarding the difference between integrated and non-integrated models is a concern. The Treatment Model Comparison Chart was used to provide definitions of integrated versus non-integrated care but was not pre-tested prior to this study. A better understanding about how to most effectively articulate the differences in treatment models to patients is important to strengthen future qualitative and quantitative studies of preferences.

## Conclusions

In this qualitative study, the majority of participants preferred non-integrated OUD treatment offered in specialty settings. Despite this, participants highlighted the convenience and the potential for treatment individualization in OUD treatment integrated into general medical settings, but they expressed concerns about privacy, the effectiveness of treatment, and a lack of access to psychosocial services. In addition, participants emphasized the importance of the patient-provider relationship. Some participants worried that general medical providers may be more judgmental, describing past negative experiences after disclosing their substance use in general medical settings. Others with strong and trusting relationships with general medical providers felt this relationship was an advantage of integrated treatment models. Overall, the findings support expanding access to both integrated and non-integrated treatment options for individuals with OUD. Recommendations to make integrated OUD treatment more patient-centered include providing or facilitating access to psychosocial services, educating patients on how their OUD treatment information will be protected within the program’s health records, incorporating approaches to allow the individualization of care, allocating time for continuing provider education on treating OUD, and prioritizing empathy and the patient-provider relationship.

## Supplementary Information

**Additional file 1: **Study interview guide.

**Additional file 2:**Treatment model comparison chart.

## Data Availability

The data that support the findings of this study are available on request from the corresponding author (ECS). The data are not publicly available due to concerns about compromising the privacy of research participants.

## Abbreviations

HIV: Human immunodeficiency virus
MOUD: Medications for opioid use disorder
mhealth: Mobile health
OUD: Opioid use disorder
PCSS-MAT: Provider Clinical Support System for Medication-Assisted Treatment
SUD: Substance use disorder
TAPS: Tobacco, Alcohol, Prescription medication, and other Substance use Tool
US: United States
