# Supplementary material for: “It’s way more than just writing a prescription”: A qualitative study of preferences for integrated versus non-integrated treatment models among individuals with opioid use disorder
Source: Addict Sci Clin Pract. 2021 Jan 27;16:8. doi: 10.1186/s13722-021-00213-1 (PMC7839299; doi:10.1186/s13722-021-00213-1)
Supplement: Supplementary file 1 — Additional file 1: Study interview guide. [file 13722_2021_213_MOESM1_ESM.docx]

**Additional file 1.** Study interview guide

1. To start off, could you tell me a little bit about your opioid use?
2. Have you ever been in treatment for your opioid use?
   1. Could you tell me about your experiences receiving treatment?
   2. Could you describe any successes or challenges you’ve experienced when you’ve received treatment in the past?
   3. What worked for you? What didn’t work?
3. Are you interested in getting treatment for your substance use right now?
   1. Why?
   2. When you think about treatment, what characteristics of the program are important to you?
      1. Do you think about the services offered, like medications, counseling, employment assistance, childcare, or transportation help?
      2. Is the location of the program important?
      3. Is your insurance coverage an important thing to consider?
      4. How about the convenience of the program?
4. (*Show informational tool for medications)* Have you heard of any medications used to treat opioid use disorders? Have you ever been prescribed any of these medications for your opioid use? Have you taken any of these on the street?
   1. (*Go down list and ask about each of the medications)* Now I’m going to ask you about what you’ve heard about these medications. What do you know about [medication]?
      1. Probes: What are some good things you’ve heard about this medication? How about some negative things?
   2. If you were going to start a medication for your opioid use, which one would you choose? If you were going to recommend a medication for opioid use to a friend, which one would you recommend?
      1. Why?
   3. What formulation (e.g., injectable, implantable, tablet, liquid) would you prefer?
      1. Why?
      2. If your doctor offered you an injectable medication, what would you say?
      3. How about an implantable medication?
   4. Would you rather take a medication every day, or take a medication less often?
      1. Why?
5. (*Show informational tool for treatment models)* People can receive treatment for their substance use in different places, including addiction treatment programs, hospitals, opioid treatment programs, or from their primary care provider. Where would you prefer to receive treatment? What model do you prefer?
   1. Do you have a primary care doctor? Would you want to receive treatment for substance use from that provider?
      1. How comfortable do you feel talking about your substance use with your primary care provider? Why?
   2. Have you ever been to the emergency room for your substance use?
      1. Would you be interested in medication for your opioid use from the emergency room?
      2. Why or why not?
6. If you could design treatment for substance use, what would it look like?
   1. What do you think are the essential ingredients to good treatment?
7. Is there anything else I didn’t ask about that you feel is important for me to know?
