# Supplementary material for: “It’s way more than just writing a prescription”: A qualitative study of preferences for integrated versus non-integrated treatment models among individuals with opioid use disorder
Source: Addict Sci Clin Pract. 2021 Jan 27;16:8. doi: 10.1186/s13722-021-00213-1 (PMC7839299; doi:10.1186/s13722-021-00213-1)
Supplement: Supplementary file 2 — Additional file 2:Treatment model comparison chart. [file 13722_2021_213_MOESM2_ESM.docx]

**Additional file 2.** Treatment model comparison chart

|  | **Integrated Treatment** | **Co-located Treatment** | **Coordinated Treatment** |
| --- | --- | --- | --- |
| **What is this treatment model?** | - Both physical health and opioid use problems are treated in the **same** setting. - The **same** provider would treat your physical health and opioid use problems. | - You would receive care for your physical health and opioid use problems in the **same** setting. - **Different** providers would treat your physical health problems and opioid use. | - You would receive care for your physical health and opioid use problems in **different** settings. - **Different** providers would treat your physical health problems and opioid use. |
| **Who would treat my opioid use problem?** | - Your doctor or team of providers. This could be your:   - Primary care provider   - HIV or hepatitis C doctor   - Maternity/   obstetric provider | - A licensed and trained addiction treatment provider. | - A licensed and trained addiction treatment provider. |
| **Where would I go to treatment for my opioid use?** | - It depends on the program. - Often, integrated treatment is offered in:   - Primary care practices   - Maternity practices   - Infectious disease (e.g., HIV or hepatitis C) practices | - It depends on the program. - Often, co-located treatment is offered in:   - Primary care practices   - Community health centers   - Hospitals | - You could go to a(n):   - Addiction treatment program   - Opioid treatment program   - Psychiatric practice |
